# Charges and Specifications

**Published:** 1864-08

**Authors:** J. Holt

**Affiliations:** Judge Advocate-General


### CHARGES AND SPECIFICATIONS

*Preferred against Brigadier-General William A. Hammond, Surgeon-General United States Army.*

CHARGE I.—"Disorders and neglects to the prejudice of good order and military discipline."

*Specification* 1*st.*—"In this: that he, Brigadier-General WILLIAM A. HAMMOND, Surgeon-General United States Army, wrongfully and unlawfully contracted for, and ordered Christopher C. Cox, as Acting Purveyor in Baltimore, to receive blankets of one William A. Stevens, of New York. This done at Washington city, on the seventeenth day of July, in the year of our Lord one thousand eight hundred and sixty-two."

*Specification* 2*d.*—"In this: that he, Brigadier-General WILLIAM A. HAMMOND, Surgeon-General as aforesaid, did on the first day of May, in the year of our Lord one thousand eight hundred and sixty-three, at Washington city, wrongfully and unlawfully, and with intent to favor private persons, residents of Philadelphia, prohibit Christopher C. Cox, as Medical Purveyor for the United States, in Baltimore, from purchasing drugs for the army in said city of Baltimore."

*Specification* 3*d.*—"In this: that he, the said Brigadier-General WILLIAM A. HAMMOND, Surgeon-General United States Army, did unlawfully order and cause one George E. Cooper, then Medical Purveyor for the

United States in the city of Philadelphia, to buy of one William A. Stevens, blankets for the use of the Government service, of inferior quality; he, the said Brigadier-General William A. Hammond, then well knowing that the blankets so ordered by him to be purchased as aforesaid were inferior in quality, and that said Purveyor Cooper had refused to buy the same of said Stevens. This done at Philadelphia, in the State of Pennsylvania, on the twenty-eighth day of May, in the year of our Lord one thousand eight hundred and sixty-two."

*Specification 4th.*—In this: that he, the said Brigadier-General William A. Hammond, Surgeon-General as aforesaid, on the fourteenth day of June, in the year of our Lord one thousand eight hundred and sixty-two, at the city of Washington, in the District of Columbia, unlawfully, and with intent to aid one William A. Stevens to defraud the Government of the United States, did, in writing, instruct George E. Cooper, then Medical Purveyor at Philadelphia, in substance, as follows:

"'Sir:—You will purchase of Mr. W. A. Stevens eight thousand pairs of blankets, of which the enclosed card is a sample. Mr. Stevens' address is box 2500, New York. The blankets are five dollars per pair;' and wh.ch blankets so ordered were unfit for hospital use."

*Specification 5th.*—"In this: that he, the said Brigadier-General William A. Hammond, Surgeon-General United States Army, on the sixteenth day of June, in the year of our Lord one thousand eight hundred and sixty-two, at the city of Washington, did corruptly, and with intent to aid one William A. Stevens to defraud the Government of the United States, give to the said William A. Stevens an order, in writing, in substance, as follows: 'Turn over to George E. Cooper, Medical Purveyor at Philadelphia, eight thousand pairs of blankets;' by means whereof the said Stevens induced the said Cooper, on Government account, and at an exorbitant price, to receive of said blankets, which he before had refused to buy, seventy-six hundred and seventy-seven pairs, and for which the said Stevens received payment at Washington in the sum of about thirty-five thousand three hundred and fourteen dollars and twenty cents."

*Specification 6th.*—"In this: that he, the said Brigadier-General William A. Hammond, Surgeon-General United States Army, on the thirty-first day of July, in the year of our Lord eighteen hundred and sixty-two, at the city of Philadelphia, in the State of Pennsylvania, well knowing that John Wyeth & Brother had before that furnished medical supplies to the Medical Purveyor at Philadelphia, which were inferior in quality, deficient in quantity, and excessive in price, did corruptly, unlawfully, and with intent to aid the said John Wyeth & Brother to furnish additional large supplies to the Government of the United States, and thereby fraudulently to realize large gains thereon, then and there give to George E. Cooper, then Medical Purveyor at Philadelphia, an order, in writing, in substance as follows: 'You will at once fill up your store-houses, so as to have constantly on hand hospital supplies of all kinds for two hundred thousand men for six months. This supply I desire that you will not use without orders from me.' And then and there directed said Purveyor to purchase a large amount thereof, to the value of about one hundred and seventy-three thousand dollars, of said John Wyeth & Brother."

*Specification 7th.*—"In this: that he, the said Brigadier-General William A. Hammond, Surgeon-General United States Army, about the eighth day of October, in the year of our Lord eighteen hundred and sixty-two, at Washington city, in contempt of, and contrary to the provisions of, the act entitled 'An act to re-organize and increase the efficiency of the Medical Department of the Army,' approved April 16, 1862, did corruptly and unlawfully direct Wyeth & Brother, of Philadelphia, to send forty thousand cans of their 'Extract of Beef' to various places, to wit: Cincinnati, St. Louis, Cairo, New York, and Baltimore, and send the account to the Surgeon-General's Office for payment; and which 'Extract of Beef' so ordered was of inferior quality, unfit for hospital use, unsuitable and unwholesome for the sick and wounded in hospitals, and not demanded by the exigencies of the public service."

*Specification 8th.*—"In this: that he, the said Brigadier-General William A. Hammond, Surgeon-General United States Army, about the *first day of March*, in the year of our Lord eighteen hundred and sixty-three, at Washington city, in diregard of his duty, of the interests of the public service, and of the requirements of the act entitled 'An act to re-organze and increase the efficiency of the Medical Department of the Army,' approved April 16, 1862, did order and direct that the Medical Inspectors should report the result of their inspections direct to the Surgeon-General."

Charge II.—"Conduct unbecoming an officer and a gentleman."

*Specification 1st.*—"In this: that he, Brigadier-General William A. Hammond, Surgeon-General United States Army, on the thirteenth day of October, in the year of our Lord eighteen hundred and sixty-three, at Washington city, in a letter by him then and there addressed to Dr. George E. Cooper, declared in substance that the said Cooper had been relieved as Medical Purveyor in Philadelphia, because, among other reasons, 'Halleck,' meaning Major-General Henry W. Halleck, General-in-Chief, requested, as a particular favor, that Murray might be ordered to Philadelphia; which declaration so made by him, the said Brigadier-General WILLIAM A. HAMMOND, Surgeon-General as aforesaid, was false."

An additional charge and specifications preferred against Brigadier-General WILLIAM A. HAMMOND, Surgeon-General United States Army:

CHARGE III.—"Conduct to the prejudice of good order and military discipline."

*Specification 1st.*—"In this: that he, the said Brigadier-General WILLIAM A. HAMMOND, Surgeon-General United States Army, on the eighth day of November, A. D. 1862, at Washington city, did, unlawfully and corruptly, order and cause Henry Johnson, then Medical Storekeeper, and Acting Purveyor at Washington city, to purchase three thousand blankets of one J. P. Fisher, at the price of $5.90 per pair, and to be delivered to Surgeon G. E. Cooper, U. S. A., Medical Surveyor at Philadelphia."

*Specification 2d.*—"In that he, the said Brigadier-General WILLIAM A. HAMMOND, about the third day of December, A. D, 1862, at Washington city, unlawfully and corruptly purchased and caused to be purchased of J. C. McGuire & Co., large quantities of blankets and bedsteads, and which were not needed for the service."

By order of the President of the United States,

J. HOLT, Judge Advocate-General.

[*Medical and Surgical Journal.*

